# Correction: Machine learning predicts pulmonary long Covid sequelae using clinical data

**DOI:** 10.1186/s12911-025-02918-8

**Published:** 2025-02-10

**Authors:** Ermanno Cordelli, Paolo Soda, Sara Citter, Elia Schiavon, Christian Salvatore, Deborah Fazzini, Greta Clementi, Michaela Cellina, Andrea Cozzi, Chandra Bortolotto, Lorenzo Preda, Luisa Francini, Matteo Tortora, Isabella Castiglioni, Sergio Papa, Diego Sona, Marco Alì

**Affiliations:** 1 Unit of Computer Systems and Bioinformatics, Department of Engineering, University Campus Bio-Medico of Rome, Via Alvaro del Portillo 21, Rome, 00128 Italy; 2 Department of Diagnostics and Intervention, Radiation Physics, Biomedical Engineering, Umeå University, Universitetstorget 4, 901 87 Umeå, Sweden; 3 Fondazione Bruno Kessler, Via Sommarive, 18, Trento, 38123 Italy; 4 Department of Physics, University of Trento, Via Sommarive, 14, Trento, 38123 Italy; 5 DeepTrace Technologies S.R.L., Via Conservatorio 17, Milan, 20122 MI Italy; 6 Department of Science, Technology and Society, University School for Advanced Studies IUSS Pavia, 27100 Pavia, Italy; 7 Department of Diagnostic Imaging and Stereotactic Radiosurgey, Centro Diagnostico Italiano S.p.A., Via S. Saint Bon 20, Milan, 20147 Italy; 8 Radiology Department, ASST Fatebenefratelli Sacco, Piazza Principessa Clotilde 3, Milan, 20121 Italy; 9 Imaging Institute of Southern Switzerland (IIMSI), Ente Ospedaliero Cantonale (EOC), Lugano, Switzerland; 10 Radiology Unit, Department of Clinical, Surgical, Diagnostic, and Pediatric Sciences, University of Pavia, Corso Str. Nuova, 65, Pavia, 27100 Italy; 11 Radiology Institute, Fondazione IRCCS Policlinico San Matteo, Viale Golgi 19, Pavia, 27100 Italy; 12 Department of Naval, Electrical, Electronics and Telecommunications Engineering, University of Genova, Via all’Opera Pia 11a, Genoa, 16145 Italy; 13 Department of Physics G. Occhialini, University of Milan-Bicocca, 20133 Milan, Italy; 14 Bracco Imaging S.p.A., Via Caduti di Marcinelle 13, Milan, 20134 Italy

**Correction to: Cordelli et al. BMC Medical Informatics and Decision Making (2024) 24:359**

10.1186/s12911-024-02745-3

Following the publication of the original article, the authors noted that some text in the ‘Results and discussion section’, including Tables 3 and 4, and 5, was from an earlier, unedited version. This occurred due to a rendering error in the software used to write the manuscript. The original article has been corrected and the changes include the removal of Tables 3, 4 and 5. The existing Table 6 has been renamed to Table 3. 

The full overview of these changes is shown in Supplementary File 1. Parts that should have been removed are marked with the red box.

The correct version of **Results and discussion** section and Table 3 are as follows:

## Results and discussion

Table 2 presents the results of the three approaches described in the previous section, one per each horizontal section of the table. By column, the table reports the model used and then the five performance scores already mentioned in terms of average and standard deviation across the cross-validation folds. Still by column, we highlight in bold the best performance attained, which reveals that classifier selection exploiting the multimodality with the SVM as meta learner returns the highest scores. It is also interesting to note that, in general, the multimodal classifier selection provides values of accuracy, specificity, and AUC that are larger than those returned by shallow machine learning and by the ensemble of learners.

To deepen the results summarized by the AUC values, and to discover possible specific regions where the high-AUC classifier might perform worse than the other low-AUC classifier, Fig. 2 plots the corresponding average ROC curves^1^. From left to right, it displays the plots of the shallow machine learning approach, of the ensemble of classifiers, and of the approach exploiting the modality selection. In the leftmost plot, we notice that the SVM curve lies over the others in a large portion of the ROC space, confirming its better performance observed in Table 2. The ROC plot in the case of ensemble learning shows that Random Forest and Majority Voting performs better than the other three approaches, since their curves lying closer to the ideal point, thus confirming the values observed in Table 2. Furthermore, while there the AUC values of the Random Forest and Majority Voting are closer, in the plot we notice that the Random Forest is more liberal than Majority Voting. The rightmost chart refers to the approach exploiting the multimodality when the model used for the selection varies: it is worth noting that the SVM lies closer to the ideal point in the ROC space, confirming its superiority to the other learners. We deem that this happens because the original feature space is in R 3 and the kernel expansion, together with the binary decomposition of the three-class classification task tackled by the model, helps obtain a linear separable space where the SVM effectively learns the boundary [48].

Finally, we focus more on the third approach exploiting the multimodality: we investigate to what extent having divided the feature set according to a medical point of view impacts the results. To this end, we randomly shuffle the features in three sets, therefore losing any medical interpretation while keeping the number of modalities for the sake of comparison. The results attained using the same selection methodology reported in Sect. 3.3 are reported in Table 3, showing that the random organization of the descriptors reduces the performance in many scores and for different models. Furthermore, in the case of the best-performing models, i.e., the SVM in both Tables 2 and 3 we found that their performance statistically differs (*p* < 0.05) according to the Wilcoxon-Mann-Whitney test.

**Table 3:** Results of the multimodal approach when the features are randomly divided. As in Table 2, missing continuous and categorical values are imputed by the mean and the mode, respectively, as reported in “Data preparation” section

Meta-learner	Performance (%)				
	Accuracy	Sensitivity	Specificity	AUC	F1-score
**Bayesian classifier** **Decision Tree** **SVM** **XGBoost**	86.4 ± 1.6 76.4 ± 1.4 **91.6 ± 0.7** 81.4 ± 0.9	69.2 ± 3.5 55.2 ± 2.9 **79.2 ± 2.6** 59.3 ± 2.1	95.8 ± 1.3 88.8 ± 1.1 **99.2 ± 0.7** 93.3 ± 1.2	92.7 ± 1.8 83.9 ± 2.1 **98.0 ± 0.5** 88.0 ± 1.7	78.3 ± 2.7 63.1 ± 2.5 **86.5 ± 1.3** 68.7 ± 1.6

## Electronic supplementary material

Below is the link to the electronic supplementary material.

Supplementary Material 1

## Footnotes

1 We decided to do not show the horizontal and vertical standard deviation to make clearer the plots.

