# Supplementary material for: Correction: Machine learning predicts pulmonary long Covid sequelae using clinical data
Source: BMC Med Inform Decis Mak. 2025 Feb 10;25:68. doi: 10.1186/s12911-025-02918-8 (PMC11812242; doi:10.1186/s12911-025-02918-8)
Supplement: Supplementary file 1 — Supplementary Material 1 [file 12911_2025_2918_MOESM1_ESM.pdf]

Cordelli et al.
BMC Medical Informatics and Decision Making (2024) 24:359
https://doi.org/10.1186/s12911-024-02745-3

BMC Medical Informatics and Decision Making

RESEARCH Open Access

Machine learning predicts pulmonary Long Covid sequelae using clinical data

Ermanno Cordelli[1], Paolo Soda[1,2*], Sara Citter[3,4], Elia Schiavon[5], Christian Salvatore[5,6], Deborah Fazzini[7], Greta Clementi[7], Michaela Cellina[8], Andrea Cozzi[9], Chandra Bortolotto[10,11], Lorenzo Preda[10,11], Luisa Francini[1], Matteo Tortora[1,12], Isabella Castiglioni[13], Sergio Papa[7], Diego Sona[3] and Marco Alì[7,14]

Abstract

Long COVID is a multi-systemic disease characterized by the persistence or occurrence of many symptoms that in many cases affect the pulmonary system. These, in turn, may deteriorate the patient's quality of life making it easier to develop severe complications. Being able to predict this syndrome is therefore important as this enables early treatment. In this work, we investigated three machine learning approaches that use clinical data collected at the time of hospitalization to this goal. The first works with all the descriptors feeding a traditional shallow learner, the second exploits the benefits of an ensemble of classifiers, and the third is driven by the intrinsic multimodality of the data so that different models learn complementary information. The experiments on a new cohort of data from 152 patients show that it is possible to predict pulmonary Long Covid sequelae with an accuracy of up to 94%. As a further contribution, this work also publicly discloses the related data repository to foster research in this field.

Keywords Long-COVID, Post-COVID syndrome, Artificial intelligence, Prognosis, Multimodal learning

*Correspondence:
Paolo Soda
paolo.soda@umu.se; p.soda@unicampus.it
[1] Unit of Computer Systems and Bioinformatics, Department of Engineering, University Campus Bio-Medico of Rome, Via Alvaro del Portillo 21, Rome 00128, Italy
[2] Department of Diagnostics and Intervention, Radiation Physics, Biomedical Engineering, Umeå University, Universitetstorget 4, 901 87 Umeå, Sweden
[3] Fondazione Bruno Kessler, Via Sommarive, 18, Trento 38123, Italy
[4] Department of Physics, University of Trento, Via Sommarive, 14, Trento 38123, Italy
[5] DeepTrace Technologies S.R.L., Via Conservatorio 17, Milan 20122, MI, Italy
[6] Department of Science, Technology and Society, University School for Advanced Studies IUSS Pavia, 27100 Pavia, Italy
[7] Department of Diagnostic Imaging and Stereotactic Radiosurgey, Centro Diagnostico Italiano S.p.A., Via S. Saint Bon 20, Milan 20147, Italy
[8] Radiology Department, ASST Fatebenefratelli Sacco, Piazza Principessa Clotilde 3, Milan 20121, Italy
[9] Imaging Institute of Southern Switzerland (IIMSI), Ente Ospedaliero Cantonale (EOC), Lugano, Switzerland
[10] Radiology Unit, Department of Clinical, Surgical, Diagnostic, and Pediatric Sciences, University of Pavia, Corso Str. Nuova, 65, Pavia 27100, Italy
[11] Radiology Institute, Fondazione IRCCS Policlinico San Matteo, Viale Golgi 19, Pavia 27100, Italy
[12] Department of Naval, Electrical, Electronics and Telecommunications Engineering, University of Genova, Via all'Opera Pia 11a, Genoa 16145, Italy
[13] Department of Physics G. Occhialini, University of Milan-Bicocca, 20133 Milan, Italy
[14] Bracco Imaging S.p.A., Via Caduti di Marcinelle 13, Milan 20134, Italy

© The Author(s) 2024. **Open Access** This article is licensed under a Creative Commons Attribution 4.0 International License, which permits use, sharing, adaptation, distribution and reproduction in any medium or format, as long as you give appropriate credit to the original author(s) and the source, provide a link to the Creative Commons licence, and indicate if changes were made. The images or other third party material in this article are included in the article's Creative Commons licence, unless indicated otherwise in a credit line to the material. If material is not included in the article's Creative Commons licence and your intended use is not permitted by statutory regulation or exceeds the permitted use, you will need to obtain permission directly from the copyright holder. To view a copy of this licence, visit http://creativecommons.org/licenses/by/4.0/.

Introduction

Since the first clinical evidence in 2019, the coronavirus disease (COVID-19) has globally affected more than 765 million people and caused nearly 7 million deaths [1, 2]. After almost four years it is still spreading worldwide and, due to its novelty, much about the clinical course remains uncertain [3]. This also applies to its long-term manifestation, which is called *Long-COVID* or *Post-COVID syndrome*. Long COVID is a multi-systemic disease characterized by the persistence or occurrence of a wide range of symptoms of varying intensity, regardless of the severity of the original illness caused by SARS-CoV-2 infection. Due to the lack of a common definition, the WHO advocated adopting the term *Post-COVID-19* listed in the ICD-10 classification based on the Delphi agreement [4]. Variable manifestations are experienced by patients with this syndrome, including fatigue, muscle soreness, palpitations, cognitive impairment, anxiety, arthralgia, and pulmonary symptoms [5]. Pulmonary post-COVID syndrome is clinically characterized by shortness of breath, even with minimal physical exertion, that can be accompanied by a decreased exercise tolerance, persistent dry or productive cough that lasts beyond the acute phase of the illness, bronchial hyperreactivity, with episodes of wheezing, chest tightness, and difficulty breathing [6]. Moreover, pulmonary fibrosis has been described as a possible complication of COVID-19 infection, with a long-term impact on patients' respiratory health, with reduced lung function and impaired oxygen exchange [7]. Post-COVID syndrome is not necessarily related to the severity of COVID-19 [8], and it is estimated that more than 17 million people are suffering from it [9]. In this context, being able to predict its development is a very ambitious challenge [10] because early treatment of the disease might reduce its impact on affected patients [11]. Nevertheless, the widespread uncertainty about the clinical utility and dosimetric appropriateness of radiological follow-up of the evolution of lung involvement by COVID-19, subject to the diffuse practice of performing chest X-rays or CT scans immediately preceding hospital discharge, has resulted in a scarce availability of extensive imaging case histories in patients recovered from COVID-19 disease or in patients with post-COVID syndrome [12]. There is therefore a need for effective and reliable approaches to predict syndrome evolution in its early stages.

Artificial Intelligence (AI) applications in medicine are widely spread among all possible fields of applications, ranging from medical image analysis to inspection of patients' clinical data for diagnosis purposes [13]. In particular, a huge number of publications appeared on the COVID-19 outbreak [14, 15], where the vast majority of works focused on the diagnosis of the COVID-19 outcome mostly starting from radiological images, such as chest X-ray images or chest CT scans, using deep-learning methods [16]. In several other papers, other machine-learning algorithms, such as Decision Trees, Random Forests and Logistic Regression showed their usefulness for prediction purposes on clinical and laboratory data. However, only a few studies investigated AI applications predicting Long-COVID syndrome [17–22]. In [17] the authors focuse on developing machine learning models to predict the incidence of Long COVID using electronic health record (EHR) data collected from the National COVID Cohort Collaborative (N3C), addressing the challenge training two machine learning models, namely logistic regression (LR) and random forest (RF), using several features from EHR data, including symptoms experienced during acute infection, medications administered, demographic information, and pre-existing health conditions. Labeling and analysing a cohort of more than 2000000 individuals as having Long COVID based on the U09.9 ICD10-CM code, they found a promising result of LR and RF models achieving median AUC values of 0.76 and 0.75 respectively. Finally, they used SHapley Additive exPlanations methods to help underscore various important predictive features. Tang C.Y. et al., in their work [18] focused on the development predictive models to assess multiple outcomes associated with COVID-19 as intensive care unit (ICU) admission, hospitalization, and the risk of Long COVID. They utilised a retrospective cross-sectional study, analysing data from 4,450 individuals who tested positive for SARS-CoV-2 collected over 2 years, and including disease courses, urban-rural classifications, demographic data, and clinical histories. The study aimed to create personalized risk assessment models that can predict clinical outcomes and forecast hospitalization, ICU admission, and Long COVID. They demonstrated the capability to identify patients at higher risk for adverse outcomes associated with COVID-19. The authors in [19] also integrated physiological and neurological factors and utilised advanced machine learning methods to investigate the health complications experienced by individuals after they recovered from COVID-19. The study was addressed by collecting survey data from COVID-19 recovered patients in Bangladesh, focusing on determining which factors most significantly impact post-COVID health outcome by using several machine learning algorithms. The study identified 17 key physiological and neurological health factors and the Decision Tree model was highlighted as particularly effective, also evaluating the statistical robustness validating the results using Chi-square tests and Pearson's coefficient methods. In [20] the authors presented a machine-learning approach based on XGBoost models, to highlight different risk factors

that appear to be most significant in predicting whether a COVID-positive person will go on to develop the long COVID syndrome. Risk factors such as chronic illnesses like diabetes, chronic kidney disease, and chronic pulmonary disease, were included, as well as non-respiratory symptoms like sleep disturbances, chest pain, and malaise after the acute COVID infection has subsided. The approach was tested on a private dataset accounting for 946 patients, and the best-developed model obtained an AUROC value of 0.92. Kessler et al. [21] used a gradient-boosting classifier (LGBM) instead. The model was trained on 272588 patients from a private dataset, 5440 of whom had a long COVID diagnosis. Age, sex, and the complete history of diagnoses and prescription data before COVID-19 infection were used as training features. On the test set the model scored 0.84 as AUC. Other authors in [22] applied random forest and LASSO models on data from a private dataset including 4182 patients, 558 of whom reported long COVID symptoms. Both models scored an AUC of 0.77. Furthermore, in [23] the authors presented a large dataset assessing the risk factors for long-term physical and psychosocial health consequences following COVID-19 diagnosis. Data were collected through direct observation and/or reviewing and extracting electronic health records or patient registries, and follow-up data were gathered using self-administered questionnaires. In the same direction, the National Institute for Health and Care Excellence, the Scottish Intercollegiate Guidelines Network, and the Royal College of General Practitioners have recently developed a guideline with a living approach, i.e. subject to periodic updates, to provide recommendations on the treatment of post-COVID syndrome [24].

On these grounds, this study investigates three AI-based approaches to predict the development of lung alteration on CT during long COVID syndrome, using clinical and laboratory data collected at the time of hospitalization. This may help in selecting patient with long COVID syndrome that deserve a closer radiologic surveillance. The first approach is based on shallow machine learning classifiers, the second works with an ensemble of classifiers, and the third makes use of classifier selection driven by a-priori medical knowledge of the type of features. The three methods presented in this study demonstrate effective strategies for predicting Long COVID, even when the patient sample size is limited. Notably, the final approach yields promising results, achieving a predictive accuracy that surpasses leading models in the field. This finding highlights how clinical data collected during the hospitalisation period can significantly inform the assessment of potential Long COVID development. As a further contribution, this work introduces a novel dataset including clinical data from 152 patients with COVID-19 who were hospitalized in four hospitals in Italy. To each patient, we associated prognostic information related to having or not of pulmonary sequelae at chest CT within 12 months after the hospital discharge. Our study therefore offers a first quantitative analysis of this new repository that can be used by other researchers and practitioners as a baseline reference.

The rest of the manuscript is organized as follows: the next section presents the materials and data preparation, “Methods” section describes the methodology. “Results and discussion” section presents and discusses the results, while “Conclusion” section offers concluding remarks.

Materials

Study design

This study includes the clinical data collected in three Italian hospitals (ASST Fatebenefratelli Sacco, Fondazione IRCSS Policlinico San Matteo, Centro Diagnostico Italiano) at the time of hospitalization and follow-up of COVID-19 patients from March 2020 to September 2021. These hospitals are located in the north of Italy and such data was generated during the clinical activity with the primary purpose of managing COVID-19 patients within the daily practice. During the period of this study, different SARS-CoV-2 variants were predominant in Italy: the original Wuhan strain and D614G in 2020, followed by the Alpha variant (B.1.1.7) in early 2021, and the Delta variant (B.1.617.2), which became dominant from May 2021 onward. All data were retrospectively reviewed and collected, after patients’ anonymization. For the data analysis, we randomly assigned to each center a symbolic label, from A up to C.

The inclusion criteria for this study were: adult patients, confirmed SARS-CoV-2 infection via RT-PCR test, and signed informed consent to participate [25]. Patients were excluded if they were younger than 18 years old, did not provide informed consent, or if their data were incomplete for the study’s clinical outcome analysis. Moreover, we reviewed RT-PCR test results and clinical records from the hospitals involved in the study to identify any episodes of COVID-19 reinfection. Patients with confirmed reinfection during the 12-months follow-up period were excluded from the final analysis to ensure that the observed pulmonary sequelae were attributable to the initial infection.

We selected these comorbidities, cardiovascular disease, neurological disease, oncological disease, diabetes, and obesity, as they are associated with an increased risk of death in Sars-Cov2 patients in a large epidemiological survey [26] and according to the clinical outcome, each of the 152 patients included in this study was assigned to two groups according to the presence or absence of

pulmonary sequelae at chest CT within 12 months after the hospital dismision. In the following text, we named as positive the class including patients with pulmonary sequelae, and negative otherwise. The positive and negative class accounts for 97 and 55 samples, respectively. The presence of pulmonary sequelae were defined as the presence of abnormalities observed in chest CT scans, including ground-glass opacities, reticulations, consolidations, or evidence of fibrosis, as described in the relevant literature [27]. These findings were confirmed by an expert radiologist with over 5 years of experience, and any doubtful cases were resolved by consensus.

Data preparation

Data underwent three pre-processing steps. First, we verified the anomalous data and outliers with our clinical partners. Outliers were defined as values falling outside the expected clinical range or identified using the interquartile range (IQR) method, which calculates the range between the first (25th percentile) and third quartiles (75th percentile) to determine thresholds for outliers. Necessary corrections were made on the basis of this revision, considering these outliers as missing values and imputing them.

Second, we removed all the features with more than 50% missing entries, and Table 1 reports the available descriptors. The values of the missing data of the remaining features were imputed instead, using the mean, for continuous variables, or the mode, in case of categorical variables. Furthermore, it is important to note that the computation of the mean or the mode values for each feature was performed considering only the training data stage of each algorithm tested. Straightforwardly, no bias was introduced since this procedure was applied respecting the training-validation-test split described in "Models validation" section. It is worth noting that the presence of missing entries in the clinical data mostly depends upon the procedures carried out in the individual hospitals as well as upon the pressure due to the overwhelming number of patients hospitalized during the COVID-19 emergency.

Third, the values of binary variables (e.g., sex) were all coded as 0 and 1, homogenized to a coherent coding, such as 0 and 1 values for comorbidities and sex, and categorical features encoded into a binary representation with One-Hot Encoding.

Methods

We investigated three AI-based approaches to predict if COVID-19-positive patients will develop pulmonary sequelae, using the clinical features presented in "Materials" section, also aiming to offer researchers and practitioners a reference baseline to process the data available within this repository. Two of these approaches leverage well-known methodologies, i.e., namely shallow machine learning and ensemble of learners; the third one is based on a multimodal approach exploiting the intrinsic multimodality of the clinical data we collected. The next three subsections present each approach, graphically summarized in Fig. 1. The fourth subsection puts these approaches in the context of multimodal learning, whilst the fifth one details the adopted validation procedures to foster further research allowing easy and fair comparison of the results, thus recommending others to measure models' performance at least as reported here.

All analyses were carried out using Python version 3.8.10, leveraging the following main libraries: NumPy (version 1.24.2), Pandas (version 1.5.3), and Scikit-Learn (version 1.2.1).

First approach: shallow machine learning

We set up this approach to build a baseline for the next experiments by implementing a paradigm that works with some classic machine learning models (top portion of Fig. 1). We considered the following classifiers: the k-Nearest Neighbor (kNN) as an instance-based classifier, the Support Vector Machine (SVM) as a kernel machine, and CART, Classification And Regression Tree, as a decision tree (DT). Each of these algorithms has been widely applied to classification tasks across domains, including medical clinical data [28–30], due to their robustness and versatility to model complex and various data distributions. We included them in our experiments to establish a solid and well-understood baseline for comparison. Notably, all three algorithms can capture non-linear relationships, a crucial feature given the complexity and potential interactions within our dataset.

Feature selection is the first step of this approach, performed using the method described in "Data preparation" section, which returned the set of retained features denoted as $F_{k,tr} \in \mathbb{R}^{n \times d}$, where k stands for the k-th cross-validation fold, tr denotes the training set of the k-th fold, n the number of training samples and d the number of considered features. $F_{k,tr}$, together with the ground truth $L_{k,tr}$, is then used to train the three shallow classifiers, whose hyper-parameters are optimized by a grid search on the validation set by maximizing the balanced accuracy, i.e., the average of recall per class since the dataset has a certain degree of imbalance. In detail, for kNN we searched for the best k-value in the interval [1, 9], for the SVM, we searched for C parameter, *gamma* and *kernel* parameter, respectively within the intervals $[10^{-1}, 10^2]$, $[10^{-4}, 1]$ and the linear, gaussian, sigmoidal and polynomial kernels, and for the DT we searched for the most performing choosing criterion between *gini* and *entropy*, the minimum number

Table 1 List of features collected for each patient, with the corresponding description

Name	Description	Overall-population (*N* = 152)	NO sequele-group (*N* = 55)	Sequele-group (*N* = 97)	Missing data	One hot encoding
Age	Patient's age (years)	63; 55-71	56; 51-65	66; 60-76	0%	No
Sex	Patient's sex (%males)	59%	44%	36%	0%	No
Cardiovascular disease	Patient had cardiovascular diseases (% reported)	46%	39%	55%	1%	No
Neurological disease	Patient had neurological diseases (% reported)	5%	2%	6%	0%	No
Oncology disease	Patient had oncological diseases (% reported)	11%	15%	8%	1%	No
Diabetes	Patient had diabetes (% reported)	17%	17%	17%	2%	No
Obesity	Patient had obesity (% reported)	20%	15%	23%	1%	No
Dyspnea	Patient had intense tightening in the chest, air hunger, difficulty breathing, breathlessness or a feeling of suffocation (%yes)	46%	35%	52%	1%	No
Smoker	Smoker (yes/no)	13%	16%	11%	1%	No
Days of fever	Days of fever up to admission (days)	6; 2.75-8.25	4; 3-7	7; 2.25-9.75	34%	No
LDH at the time hospital admission	Lactate Dehydrogenase (mU/ml)	335; 263.5-447.0	279; 221.5-343.5	367.5; 298.25-490.75	11%	No
CRP at the time of hospital admission	C-Reactive Protein (mg/dL)	12.6; 5.3-25.6	8.59; 4.89-31.2	15.2; 6.53-24.80	4%	No
D-dimer at the time of hospital admission	D-dimer is a protein fragment that's made when a blood clot dissolves in the body (ng/mL)	640; 308.5-1105.5	426; 208.5-761	751; 360-1249.5	14%	No
Creatinine at the time of hospital admission	Creatinine is a waste product that comes from the normal wear and tear on muscles of the body (mg/dL)	0.86; 0.69-1.09	0.85; 0.65-1.02	0.88; 0.71-1.13	9%	No
CRP at the time of hospital dismission	Value of CRP at the time of dismission (mg/dL)	0.82; 0.2-2.98	0.89; 0.24-2.91	0.82; 0.19-3.1	32%	No
Creatinine at the time of hospital dismission	Value of Creatinine at the time of dismission (mg/dL)	0.74; 0.62-0.95	0.8; 0.69-0.93	0.74; 0.61-0.95	30%	No
SpO_2 at the time of admission	Value of the oxygen saturation of the arterial blood at the time of admission (%)	0.93; 0.91-0.96	0.95; 0.93-0.98	0.93; 0.88-0.95	18%	No
O_2 at the time of admission	Value of the oxygen consumption at the time of admission (%)	0.76; 0.59-0.91	0.78; 0.67-0.93	0.70; 0.58-0.90	27%	No
Days of hospitalization	Number of days the patient was on ventilation	22; 14-35	14; 11-23	29; 20-40	6%	No
Ventilation	Presence of ventilations	46%	36%	64%	0%	Yes
XR at the time of hospital admission	Chest XR acquisition at the time of hospital admission (yes/no)	90%	92%	87%	0%	No
CT at the time of hospital admission	Chest CT acquisition at the time of hospital admission (yes/no)	53%	43%	69%	0%	No
XR at the time of hospital dismission	Chest XR acquisition at the time of hospital dismission (yes/no)	38%	39%	35%	0%	No

Table 1 (continued)

Name	Description	Overall-population (*N* = 152)	NO sequele-group (*N* = 55)	Sequele-group (*N* = 97)	Missing data	One hot encoding
CT at the time of hospital dismission	Chest CT acquisition at the time of hospital dismission (yes/no)	29%	33%	22%	24%	No

of sample leaves in [1, 2, 3, 10] and the minimum number of sample splits in [1, 2, 10, 100].

Second approach: an ensemble of learners

It is well known that an ensemble of multiple learners allows extracting complementary and more powerful data representation, to improve the performance concerning those of each stand-alone classifier considered. Although no formal proof exists, this intuition has brought interesting results in many medical applications [31–34]. There are six main strategies to generate a diverse pool of classifiers [35]: the use of different initialization, different parameters, different architectures, different classifier models, different training sets, and different feature sets. In our application, as depicted in the central portion of Fig. 1, we investigated different approaches for combining single classifiers. They are: *late fusion*, *stacking*, *boosting* and *random forest*.

Late fusion approaches combine different trained models using an aggregation function that takes as input the individual predictions and selects the output label of the samples on the basis of the majority-voted classes. Despite its simpleness, this rule has provided good performance in several applications, especially when the base classifiers are diverse, i.e., when they provide different outputs on the same samples, thus offering complementary points of view to the ensemble since there is no reason to combine models that always return the same output [36].

The stacking approach determines the final output for a given sample by using a classifier trained on the predictions provided by the base classifiers. Hence, it can be considered a late fusion technique where a data-driven aggregation rule is used: indeed, it is not heuristically set by the researcher, but it is learned by a model.

In the case of majority voting and stacking, we aggregated in an ensemble the three classifiers mentioned in the previous subsection.

Boosting is a well-established ensemble meta-algorithm that converts weak learners to strong ones during the training process. Here we adopted the AdaBoost approach with two variations as a base estimator: the first uses the ExtraTree, whilst the second employs the

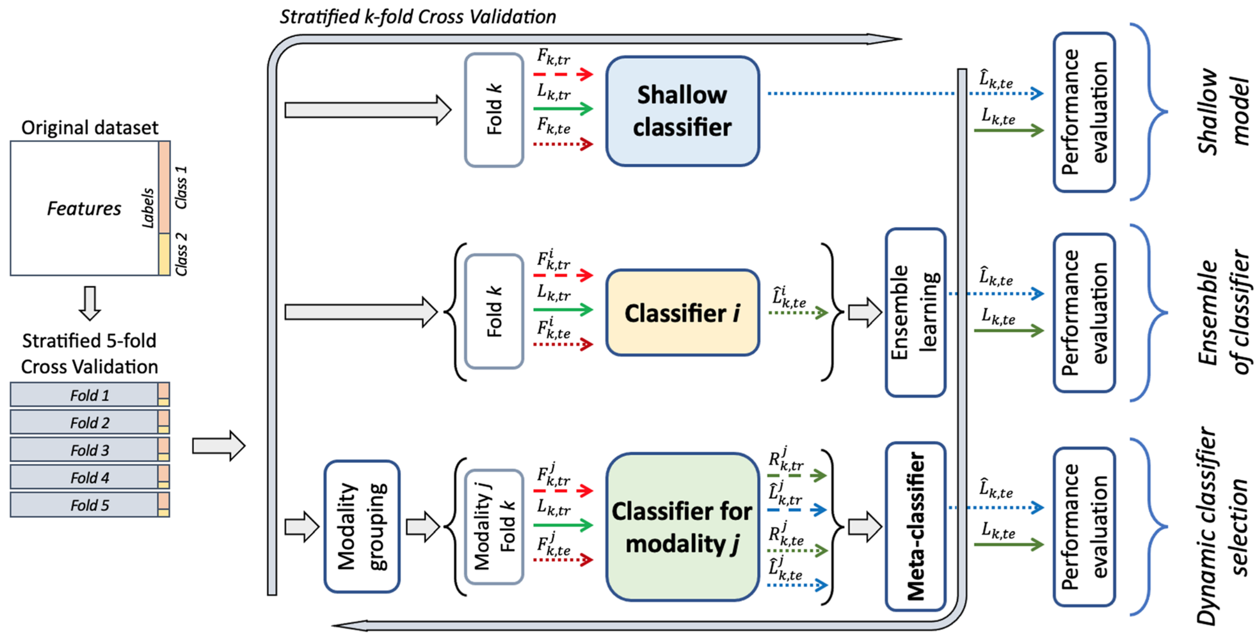

Fig. 1 Graphical representation of the methodology with the three different approaches. Symbols are defined in "Exploiting the multimodality: classifier selection" section

logistic regression and L2 regularization. These models had shown good classification capabilities in both assessing the presence of the COVID-19 infection [37] and mortality risk [38]. When using the ExtraTree, we optimized, via a randomized search, the following hyperparameters during the training phase: the number of estimators (sampled from a log uniform distribution between 50 and 1000), the learning rate (sampled from a log uniform distribution between 0.0001 and 10), the minimum samples per leaf (sampled from a log uniform distribution between 1 and 10), the minimum samples for splitting (sampled from a uniform distribution between 0.1 and 0.9), and class weights to compensate for the class imbalance. For this last parameter we tried: a balanced approach that adjusts weights inversely proportional to class frequencies in the input data as $n_{samples}/(n_{classes} * n_{samples\_per\_class}(y))$, a pre-established multiplier for the minority class set to 2, 3 and 5. When using logistic regression and L2 regularization, we optimized the number of estimators, the learning rate, the magnitude of the regularization term, and again the class weights assigned to the two classes by the base estimator. The searched range for parameters is the same as those specified for the ExtraTrees case, except for the strength of the regularization term, whose inverse was sampled from a log uniform distribution in the range 0.0001 and 1000. Furthermore, to eliminate non-informative features, improve performance and reduce the training time, we applied the boosting methods not only to the whole set of features, but also to a set of features restricted, excluding high-correlated and near-zero variance features (manual feature selection), and further reduced by Recursive Feature Elimination [39], from a maximum of 10 to a minimum of 4.

As a fourth ensemble paradigm, we used the Random Forest approach that constructs a multitude of decision trees at training time. It is a well-known ensemble method that combines the output of multiple decision trees to reach a single result [40]. Furthermore, we did not run feature selection in this case thanks to the intrinsic ability of the decision trees to detect the most important descriptors.

Exploiting the multimodality: classifier selection

The use of a feature space including all the available descriptors can hinder peculiarities that should be included in specific groups of features. This third learning approach stems from this observation, and it also exploits that the collected features can be divided into three different modalities from a medical point of view. Each modality groups the features as follows:

- Modality 1: Anamnestic data, which includes Age at hospitalization, Sex, Cardiovascular disorders, Neurological disorders, Oncological disorders, Diabetes, Obesity, Dyspnea and Smoking attitude;
- Modality 2: Hospitalisation data, which includes X-Ray at hospitalization, unenhanced chest CT at hospitalization, Days of fever at hospitalization, Lactate Dehydrogenase (LDH) at hospitalization, C Reactive Protein (CRP) at hospitalization, D-dimer at hospitalization, Creatinine at hospitalization, X-Ray at discharge, unenhanced chest CT at discharge, CRP at discharge and Creatinine at discharge;
- Modality 3: Ventilation data, which includes Peripheral Oxygen Saturation (SpO_2) at hospitalization, Oxygen Saturation (O_2) at hospitalization, Days of ventilation, Continuous positive airway pressure (CPAP) ventilation and Non-invasive ventilation (NIV) ventilation.

Such three modalities fed three different classifiers, one per modality, constructing decision boundaries in a modality-specific feature space. It is worth noting that such an approach exploits one of the strategies to generate a diverse pool of classifiers mentioned in the previous subsection, i.e., the use of different feature sets that is the more successful in generating base experts that are more diverse and informative [41]. However, different from the methods mentioned in "Second approach: an ensemble of learners" section, here we define an approach that selects the most competent classifier, a step that can be conducted either in a static or dynamic fashion. In static selection methods, the expert is selected during the training phase, according to a selection criterion estimated on the validation set, and it will be used to predict the label of all samples in the test set. Oppositely, in the dynamic selection, the most competent classifier is selected specifically to classify each unknown example, thus exploiting the fact that each base classifier is an expert in distinct regions of the feature space.

Our approach works with dynamic selection and, according to the taxonomy proposed [35], our selection criterion belongs to meta-learning. Indeed, we introduce a meta-classifier that, for each input sample, specifies which is the classifier that has to be used to set the final label among the three available. This meta-learner receives as input the classification reliabilities provided by the three base classifiers. Let us recall that classification reliability, commonly referred to as confidence or credibility, is a measure in [0, 1] computed using information directly derived from the output of the expert, by establishing a correspondence between the expert's output and the sample situations in the feature space [42]. This allows taking into account the issues

influencing the achievement of satisfactory results, such as the noise affecting the samples domain or the difference between objects to be recognized and those used to train the classifier [43].

Formally, our selection approach works in the training and testing phases as follows (bottom portion of Fig. 1):

- For each fold, $F_{k,tr}$ and $F_{k,te}$ (where *te* stands for testing) is further subdivided by grouping the features according to the three modalities described before, denoted by m_j, with $j = 1, 2, 3$, obtaining $F^j_{k,tr}$ and $F^j_{k,te}$;
- The training features $F^j_{k,tr}$ together with the ground truth $L_{k,tr}$ are then used to train the expert e_j on the modality m_j;
- After training, each expert e_j provides $\hat{L}^j_{k,tr} \in \mathbb{R}^{n\times1}$ and $R^j_{k,tr} \in \mathbb{R}^{n\times1}$, which are the predicted labels and the reliabilities for all the training samples;
- The meta-classifier is trained using $\left\{R^j_{k,tr}\right\}^3_{j=1}$, i.e., a matrix of size $n \times 3$ concatenating the reliabilities returned by each expert, and using as ground truth a vector specifying which classifier works correctly for each training sample. In the case of multiple correct classifiers for a training sample, we specified the one with the largest reliability.
- During the test phase, each expert e_j receives $F^j_{k,te}$ and provides both $\hat{L}^j_{k,te}$ and $R^j_{k,te}$;
- Still in the test phase, the meta-learner receives $R^j_{k,te}$ and outputs the index of the expert to be used to set the final decision. Formally:

$$\hat{L}_{k,te} = \hat{L}^{j^*}_{k,te}, \text{ with } j = \{1, 2, 3\} \quad (1)$$

  and j^* the index of the selected modality.

For the sake of completeness, let us report that we used the Random Forest as a base expert per each modality, whereas we considered a pool of different models as a meta-classifier. This pool includes a Bayesian classifier, a DT, an SVM, and the XGBoost, using their default hyperparameters.

The three approaches in the context of multimodal learning

Before delving into model validation, here we discuss how the three approaches mentioned so far fit the context of multimodal learning. According to the definition offered in [44], multimodal learning combines data from different modalities of a common phenomenon, each providing separate views, to solve an inference problem. In our case, the availability of different information for a patient provides us the opportunity to take advantage of such rich information. Techniques for multimodal data fusion have been investigated by the research biomedical community [44–46]. Traditionally, there exist three main strategies to merge different modalities' information: early, late, and joint fusion. In the first technique, the features of each modality are merged according to a rule into a feature vector to be given to the learner, eventually removing correlations between modalities or representing the fused data in a lower-dimensional common subspace. In the second, the predictions provided by different learners, even one per modality, are aggregated by an aggregation rule. The third approach is designed for (deep) neural networks since the different modalities are fused at different levels of abstractions offered by the hidden layers. Although deep-learning-based multimodal learning should offer several advantages over conventional machine-learning methods [45], it needs a certain amount of training data that are not available in this application. Furthermore, machine learning methods still outperform deep learning approaches on real tabular datasets, as experimentally demonstrated in [47].

According to this taxonomy, shallow learning ("First approach: shallow machine learning" section) is a form of early fusion, whereas the approach leveraging the ensemble of classifiers ("Second approach: an ensemble of learners" section) is a hybrid between early and late fusion. Indeed, while each base classifier is trained on all the features, the overall approach benefits from the diversity given by the use of several classifiers. The method that exploits the multimodality according to a medical-based data split ("Exploiting the multimodality: classifier selection" section) belongs to late fusion because each learner is trained with a different modality, and the decisions are then combined in the selection stage.

Models validation

Model validation for the three approaches consists of 5-fold stratified cross-validation approach, which preserves the original imbalance between the classes among all the folds. For each cross-validation run, the training set is composed of four-folds, extracting the validation set for data normalization, parameters' estimation and/or features' selection depending on the applied approach, as detailed before. Straightforwardly, the test set is composed of one fold and it is used to assess the performance. To improve the statistical robustness, the entire procedure was repeated 10 times.

With reference to the measured performance score, we considered the accuracy, sensitivity, specificity, Area Under the ROC Curve (AUC), and F1-score.

Table 2 Results of the three proposed approaches when missing continuous and categorical values are imputed by the mean and the mode, respectively, as reported in "Data preparation" section

Method		Performance (%)				
		Accuracy	Sensitivity	Specificity	AUC	F1-score
**Shallow Machine Learning**	**kNN**	72.7 ± 3.2	81.9 ± 3.3	56.5 ± 7.8	74.0 ± 5.2	79.3 ± 2.3
	**SVM**	77.0 ± 4.8	81.6 ± 5.1	68.9 ± 5.3	80.6 ± 2.1	81.9 ± 4.0
	**DT**	65.9 ± 5.4	73.2 ± 11.2	52.9 ± 9.1	63.1 ± 4.0	73.1 ± 5.8
**Ensemble classification**	**Majority Voting**	74.2 ± 5.7	84.5 ± 7.1	56.0 ± 9.2	79.4 ± 5.3	80.7 ± 4.6
	**Stacking**	74.1 ± 5.0	81.4 ± 6.4	61.1 ± 6.5	76.1 ± 2.4	80.0 ± 4.3
	**AdaBoost**[a]	70.7 ± 1.5	73.1 ± 2.9	66.2 ± 3.8	73.6 ± 1.6	76.2 ± 1.5
	**AdaBoost**[b]	72.1 ± 1.8	71.4 ± 2.9	73.6 ± 2.0	76.1 ± 1.9	76.6 ± 1.9
	**Random Forest**	77.6 ± 2.4	**89.2 ± 4.3**	57.1 ± 6.0	81.4 ± 3.1	83.5 ± 2.0
**Multimodal approach**[c]	**Bayesian classifier**	87.4 ± 1.5	72.9 ± 2.5	95.7 ± 1.6	93.1 ± 1.2	80.8 ± 2.3
	**Decision Tree**	82.4 ± 1.3	67.5 ± 3.0	90.9 ± 1.6	89.6 ± 1.4	73.4 ± 2.5
	**SVM**	**94.6 ± 1.0**	87.3 ± 2.3	**98.8 ± 0.8**	**98.0 ± 0.4**	**92.1 ± 1.5**
	**XGBoost**	84.5 ± 1.3	68.2 ± 4.4	93.8 ± 2.0	91.8 ± 1.3	76.0 ± 2.4

[a] ExtraTree as base estimator

[b] Logistic regression and L2 regularization as base estimator

[c] Only meta learner, Random Forest as base classifier

Results and discussion

Table 2 presents the results of the three approaches described in the previous section, one per each horizontal section of the table. By column, the table reports the model used and then the five performance scores already mentioned in terms of average and standard deviation across the cross-validation folds. Still by column, we highlight in bold the best performance attained, which reveals that classifier selection exploiting the multimodality with the SVM as meta learner returns the highest scores. It is also interesting to note that, in general, the multimodal classifier selection provides values of accuracy, specificity, and AUC that are larger than those returned by shallow machine learning and by the ensemble of learners.

To deepen the results summarized by the AUC values, and to discover possible specific regions where the high-AUC classifier might perform worse than the other low-AUC classifier, Fig. 2 plots the corresponding average ROC curves[1]. From left to right, it displays the plots of the shallow machine learning approach, of the ensemble of classifiers, and of the approach exploiting the modality selection. In the leftmost plot, we notice that the SVM curve lies over the others in a large portion of the ROC space, confirming its better performance observed in Table 2. The ROC plot in the case of ensemble learning shows that Random Forest and Majority Voting performs better than the other three approaches, since their curves lying closer to the ideal point, thus confirming the values observed in Table 2. Furthermore, while there the AUC values of the Random Forest and Majority Voting are closer, in the plot we notice that the Random Forest is more liberal than Majority Voting. The rightmost chart refers to the approach exploiting the multimodality when the model used for the selection varies: it is worth noting that the SVM lies closer to the ideal point in the ROC space, confirming its superiority to the other learners. We deem that this happens because the original feature space is in $\mathbb{R}^3$ and the kernel expansion, together with the binary decomposition of the three-class classification task tackled by the model, helps obtain a linear separable space where the SVM effectively learns the boundary [48].

Let us now turn our attention to Table 3, which presents the results attained using different imputation approaches. In the first column, *baseline mean* stands for the approach presented in "Data preparation" section, where the continuous and categoric missing descriptors are imputed by the mean and mode, respectively; *mode* and *median* indicates that we imputed all missing values using the mode or the median of the corresponding feature; *1NN* denotes the imputation performed by 1NN, after having applied one-hot encoding to all categorical variables. The Friedman test performed for each performance metric does not detect significant differences between the different imputation strategies (p-value > 0.05), suggesting that the baseline approach presented

[1] We decided to do not show the horizontal and vertical standard deviation to make clearer the plots.

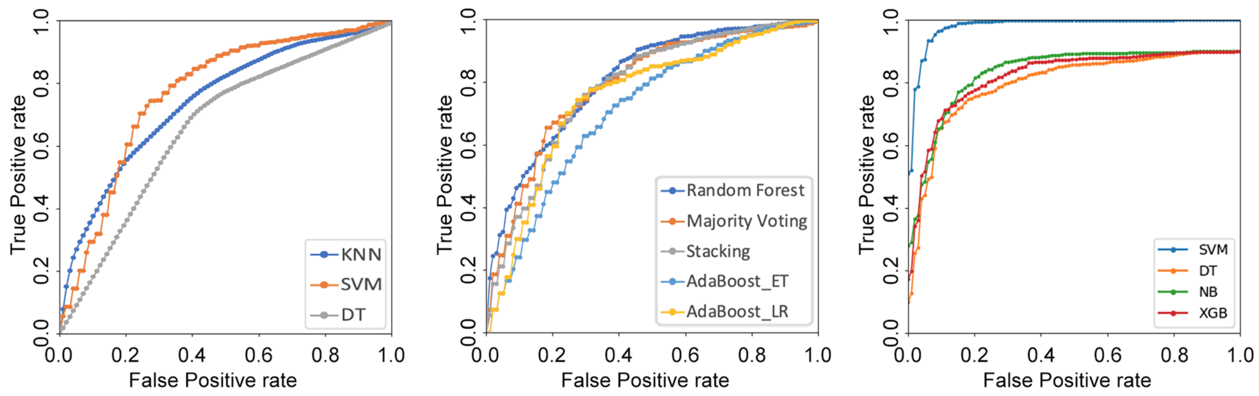

Fig. 2 ROC plots of the three approaches. From left to right, it displays the plots of the shallow machine learning approach, of the ensemble of classifiers and of the approach exploiting the modality selection

Table 3 Accuracy, F1-score, and AUC comparison among different imputation strategies. The values are the mean of the results after each repetition, with the 95% CI. In bold the best results per metric

Model	Imputation strategy	Performance (%)				
		Accuracy	Sensitivity	Specificity	AUC	F1-score
kNN	**Baseline Mean**	72.7 ± 3.2	81.9 ± 3.3	56.5 ± 7.8	74.0 ± 5.2	79.3 ± 2.3
	**Mode**	70.8 ± 4.7	79.3 ± 7.6	55.8 ± 5.2	71.8 ± 3.9	77.6 ± 4.4
	**Median**	72.0 ± 5.4	**82.8 ± 5.8**	53.1 ± 8.4	72.3 ± 4.8	79.1 ± 4.2
	**1NN**	**74.0 ± 4.9**	80.8 ± 5.9	**62.0 ± 5.8**	**75.7 ± 3.5**	**79.8 ± 4.2**
SVM	**Baseline Mean**	**77.0 ± 4.8**	**81.6 ± 5.1**	**68.9 ± 5.3**	**80.6 ± 2.1**	**81.9 ± 4.0**
	**Mode**	76.1 ± 3.0	80.6 ± 3.6	68.2 ± 3.1	80.4 ± 2.6	81.2 ± 2.6
	**Median**	76.4 ± 3.3	80.7 ± 3.9	68.9 ± 4.0	80.5 ± 2.1	81.4 ± 2.8
	**1NN**	75.8 ± 4.9	80.8 ± 6.2	66.9 ± 5.4	80.1 ± 1.9	81.0 ± 4.3
DT	**Baseline Mean**	65.9 ± 5.4	73.2 ± 11.2	52.9 ± 9.1	63.1 ± 4.0	73.1 ± 5.8
	**Mode**	65.7 ± 4.6	72.3 ± 7.7	54.0 ± 11.6	63.1 ± 5.2	72.8 ± 4.2
	**Median**	**67.2 ± 6.4**	**74.5 ± 7.1**	**54.4 ± 13.3**	**64.4 ± 7.3**	**74.4 ± 5.2**
	**1NN**	65.4 ± 7.2	73.2 ± 11.5	51.8 ± 9.0	62.5 ± 6.3	72.9 ± 7.1

before is a viable alternative also for ensemble learning and multimodal selection.

With regards to the feature selection methods described in “Second approach: an ensemble of learners” section, results from excluding high-correlation and near-zero variance features (manual feature selection) are reported in Table 4. The features identified by this method are age, sex, cardiovascular disease, dyspnea, days of fever, LDH (mU/ml) at the time of hospital admission, O_2 value (%) at the time of hospital admission, CRP value (mg/dL) at the time of hospital admission, D-dimer value (ng/mL) at the time of hospital admission, ventilation, and days of hospitalization. Interestingly, the performance of AdaBoost algorithms improves with this selection, which is not the case for the other shallow and ensemble methods implemented. However, when RFE is applied before training AdaBoost, there is no improvement in the classification performance, as can be seen in Table 5. As a result, we decided not to apply RFE to the other ensemble methods proposed. Table 5 solely reports the results of the best-performing AdaBoost algorithm with respect to the different feature-selection methods used, which, in all our experiments, turned out to be the one that uses logistic regression as the base estimator (also see Tables 2 and 4).

Finally, we focus more on the third approach exploiting the multimodality: we investigate to what extent having divided the feature set according to a medical point of view impacts the results. To this end, we randomly shuffle the features in three sets, therefore losing any medical interpretation while keeping the number of modalities for the sake of comparison. The results attained using the same selection methodology reported in “Exploiting the multimodality: classifier selection” section are reported

Table 4 Risultati solo dei test di "manual feature selection", metodo di imputation da usare è quello della sezione II.B

**Method**		**Performance (%)**				
		**Accuracy**	**Sensitivity**	**Specificity**	**AUC**	**F1-score**
**Shallow Machine Learning**	**kNN**	71.3 ± 3.5	79.8 ± 6.1	56.4 ± 9.5	74.7 ± 3.9	78.0 ± 3.0
	**SVM**	73.8 ± 4.4	77.6 ± 4.6	67.1 ± 5.8	77.3 ± 2.7	79.1 ± 3.7
	**DT**	68.8 ± 4.6	75.0 ± 7.5	57.8 ± 13.3	66.4 ± 5.6	75.4 ± 3.9
**Ensemble classification**	**Majority Voting**	74.9 ± 5.5	85.3 ± 6.9	56.5 ± 8.3	79.4 ± 3.2	81.2 ± 4.5
	**Stacking**	75.6 ± 4.4	85.7 ± 4.2	57.8 ± 5.9	78.4 ± 3.1	81.7 ± 3.4
	**AdaBoost (ExtraTree as base estimator)**	73.1 ± 2.2	75.2 ± 0.4	68.8 ± 3.1	76.2 ± 1.6	77.9 ± 2.4
	**AdaBoost (logistic regression and L2 regularization as base estimator)**	75.4 ± 0.8	74.5 ± 1.2	76.6 ± 1.6	78.9 ± 1.1	79.5 ± 0.8
	**Random Forest**	77.4 ± 3.4	88.9 ± 4.8	57.3 ± 6.0	81.0 ± 3.5	83.4 ± 2.7

Table 5 In this table the performance results of the AdaBoost classifier with logistic regression base estimator are shown. Each row shows one of the different method of feature selection (FS) tested. The reported values are the mean result, reported as a percentage, of each metric computed at the end of each repetition of the cross-validation, with the relative 95% CI. In bold the best results per metric

METHOD	**Accuracy**	**Sensitivity**	**Specificity**	**AUC**	**F1-score**
No FS	72.1 ± 1.8	71.4 ± 2.9	73.6 ± 2.0	76.1 ± 1.9	76.6 ± 1.9
Manual FS	**75.4 ± 0.8**	**74.5 ± 1.2**	**76.6 ± 1.6**	**78.9 ± 1.1**	**79.5 ± 0.8**
Recursive FS	74.6 ± 1.3	74.4 ± 0.2	74.6 ± 1.1	78.1 ± 1.5	78.9 ± 1.4

in Table 6, showing that the random organization of the descriptors reduces the performance in many scores and for different models. Furthermore, in the case of the best-performing models, i.e., the SVM in both Tables 2 and 6 we found that their performance statistically differs ($p < 0.05$) according to the Wilcoxon-Mann-Whitney test.

Discussion

Long COVID, or post-acute sequelae of SARS-CoV-2 infection, has hampered patient and societal recovery from the COVID-19 pandemic. Long COVID is characterized by changing, varied symptoms, making an unequivocal definition difficult to construct, with a urgent need to understand this syndrome, define therapies and early identify patients who will develop it. The pathophysiology of long COVID is complex and variable in terms of immunology, neurobiology, endocrinology, physiology [49]. It is important to note that our study was conducted during a period (March 2020 - September 2021) when the original Wuhan strain, the Alpha variant (B.1.1.7), and the Delta variant (B.1.617.2) were predominant in Italy. These strains, particularly Delta, were associated with more severe disease outcomes and higher hospitalization rates compared to the Omicron variant, which emerged after the study period. Omicron, while more transmissible, has generally been associated with milder clinical outcomes, particularly in vaccinated individuals. The relevance of our findings, particularly regarding pulmonary sequelae, may be limited when applied to the Omicron variant and future strains, given the different clinical manifestations of the disease. Future studies are needed to evaluate the impact of evolving variants on the development of Long COVID and its long-term outcomes. Despite these differences in variants, our study provides valuable insights into the long-term pulmonary sequelae of

Table 6 Results of the multimodal approach when the features are randomly divided. As in Table 2, missing continuous and categorical values are imputed by the mean and the mode, respectively, as reported in "Data preparation" section

**Meta-learner**	**Performance (%)**				
	**Accuracy**	**Sensitivity**	**Specificity**	**AUC**	**F1-score**
**Bayesian classifier**	86.4 ± 1.6	69.2 ± 3.5	95.8 ± 1.3	92.7 ± 1.8	78.3 ± 2.7
**Decision Tree**	76.4 ± 1.4	55.2 ± 2.9	88.8 ± 1.1	83.9 ± 2.1	63.1 ± 2.5
**SVM**	**91.6 ± 0.7**	**79.2 ± 2.6**	**99.2 ± 07.**	**98.0 ± 0.5**	**86.5 ± 1.3**
**XGBoost**	81.4 ± 0.9	59.3 ± 2.1	93.3 ± 01.2	88.0 ± 1.7	68.7 ± 1.6

severe COVID-19 infections, particularly for patients hospitalized during the earlier phases of the pandemic. The pathophysiology of Long COVID remains complex and variable, and our findings underscore the importance of early identification and management of patients at risk of developing long-term complications, regardless of the strain involved.

A comparison of the results obtained through the proposed method with those currently available in the literature reveals a general improvement in the accuracy of assessing the risk of developing Long COVID. This enhancement surpasses existing findings by utilizing clinical information collected from hospitals during patients' hospitalization periods. This approach offers significant advantages in terms of data collection efficiency, as these are standard pieces of information routinely gathered and monitored in clinical practice during hospitalization. Furthermore, the proposed method effectively leverages various data types typically considered upon patient admission and monitored throughout their stay, including medical history, hospitalization data, and ventilation information. From a technical perspective, it is clear that utilizing a meta-classifier designed to optimize predictions from multiple expert classifiers can effectively resolve potential conflicts in decision-making. This capability enhances the accuracy of assessing a patient's risk of developing Long COVID, leading to significant improvements over traditional methods that depend on individual classifiers. These classical approaches often demonstrate increased susceptibility to bias, particularly when working with limited datasets.

Early identification of post-COVID-19 sequelae is crucial for providing appropriate care and support to affected individuals. By identifying high-risk patients, healthcare providers can implement targeted interventions, regular follow-ups, and personalized treatment plans to mitigate the long-term effects of COVID-19 and improve patient outcomes. In this respect, by developing three AI-based approaches we have demonstrated the possibility of predicting possible pulmonary sequelae that can represent the cause of the long COVID syndrome. This can assist healthcare professionals in identifying patients who are more susceptible to developing long COVID and provide appropriate care and support to mitigate its long-term effects. More specifically, we believe that this method can lead to a twofold concrete benefit. From the physician's perspective, it can improve informed decision-making by allowing physicians to identify patients at high risk of Long COVID early in treatment. Such identification facilitates the tailored follow-up care and monitoring strategies, thus improving overall patient management. Furthermore, by predicting which patients are more likely to develop Long COVID, healthcare providers can allocate resources more effectively, ensuring that those at higher risk receive appropriate attention and interventions. Moreover, the presented approach could assist clinicians in enhancing patient communication. In fact, by providing a clear risk assessment, it fosters discussions with patients regarding their individual risk factors, emphasizing the importance of follow-up care and potential lifestyle adjustments post-discharge. The integration of a user-friendly interface-where clinicians can input demographics, medical history, and laboratory values-can further streamline workflow efficiency, allowing for rapid assessments during patient consultations. Furthermore, from the patients side, the development of such a predictive model offers the benefit receiving a quantifiable risk score for developing Long COVID based on their specific clinical data, thereby empowering them with critical knowledge about their health status. This understanding, in turn, can motivate patients to engage in proactive health management behaviors, such as adhering to follow-up appointments and implementing recommended lifestyle modifications and agree a shared decision-making with the healthcare providers, stimulating collaborative discussions regarding preventive measures and treatment options.

Nevertheless, our study presents some limitations. First, we included only patients hospitalized for COVID-19, without considering patients treated only at home. Second, the study requires further validation on diverse and larger populations to ensure its generalizability and effectiveness. The availability of more training data would give the chance to investigate deep learning-based approaches that immensely depend on the availability of large training data to reduce the risk of over-fitting. Third, due to the retrospective nature of the study, we included clinical data and laboratory tests which were most frequently collected at the time of hospitalization, but other clinical parameters, such as other comorbidities or blood tests could not be analysed. Also the radiological presentation of the disease could have been included in the investigation, even if, in the absence of updated universally recognized international guidelines on the imaging modality and timing, we preferred to avoid the inclusion of examinations associated with radiation exposure, preferring clinical and laboratory parameters. Additionally, past SARS-CoV-2 infections prior to hospitalisation were not systematically accounted for, and COVID-19 vaccination status was not consistently available for all patients. Both previous infections and vaccination status could potentially influence the development of pulmonary sequelae and Long COVID. These factors were not included in the present analysis and represent important

limitations of the study. Future studies should aim to include these variables to provide a more comprehensive assessment of Long COVID outcomes.

Finally, we acknowledge that while our current sample size is sufficient to conduct a valid analysis, it may not ensure the statistical robustness necessary to prevent generalisation bias. Consequently, we are committed to expanding the cohort size, which will not only enhance the reliability of our findings but also enable us to integrate state-of-the-art deep learning techniques into our methodology. Furthermore, the integration of an external dataset will be essential to ensure a more equitable comparison of the results. This step will enhance the validity of our findings and provide a broader context for interpretation, while improving the overall rigour and applicability of our research.

Conclusion

With over 765 million documented infections and 7 million deaths worldwide, the COVID-19 pandemic continues unabated still in fall 2023. An important aspect of the disease is the post-COVID syndrome, which is not necessarily related to the severity of the main symptoms, but still affects a large portion of the pandemic's victims. Predicting its development represents one of the most ambitious challenges, particularly with regard to pulmonary sequelae and using only clinical data collected during the patient's hospitalization.

In this context, we collected here demographic, clinical, and laboratory test data and, then, we proposed three AI-based approaches that have proved to be a valuable ally against this syndrome. They span from shallow machine learning to classifier selection driven by a medical-inspired multimodal analysis that, as an indirect observation of the results attained, confirms that feature engineering is a crucial part of tackling real-world challenges.

Future work is directed towards different issues. First, we plan to address the limitations mentioned at the end of the previous "Discussion" section, enlarging the dataset not only in terms of cardinality but also considering other **clinical parameters and possible correlation with pulmonary function at spirometry**. A second direction of future investigation is to explore potential mechanisms underlying the development of long COVID, improving also our understanding of the evolution of post-COVID-19 sequelae.

Abbreviations

kNN	k-Nearest Neighbor
SVM	Support Vector Machine
DT	Regression Tree
LDH	Lactate Dehydrogenase
CRP	C Reactive Protein
SpO_2	Peripheral Oxygen Saturation
CPAP	Continuous positive airway pressure
NIV	on-invasive ventilation
AUC	Area Under the ROC Curve

Acknowledgements
Not applicable.

Authors' contributions
E.C., P.S., S.C., D.F., A.C., I.C., S.P., D.S. and M.A. conceptualised the overall study. E.C., S.C., C.S., D.F., M.C., C.B., L.P. and M.A. organized and prepared the data for analysis. E.C., P.S., S.C., C.S., G.C. and D.S. conducted the formal analysis. S.P. and M.A. secured the necessary funding to support the research. E.C., P.S., S.C., D.F., D.S. and M.A. investigated the experiments. E.C., P.S., S.C., C.S., E.S., D.F., G.C., A.C., L.F., M.T., D.S. and M.A. designed the research methodology and procedures. P.S., I.C., S.P., D.S. and M.A. managed the overall project and coordination. P.S., S.P., D.S. and M.A. provided the necessary recources and materials. E.C., S.C., C.S., E.S., G.C., L.F., M.T. and D.S. developed the software for the project. P.S., D.S. and M.A. supervised the research team. E.C., P.S., S.C., C.S., E.S., D.F., D.S. and M.A. valdidated the results. E.C., P.S., S.C., C.S., E.S., D.S. and M.A. created the graphical representations of the data. E.C., P.S., S.C., E.S., G.C., M.C., D.S. and M.A. authored the initial manuscript draft. E.C., P.S., S.C., E.S., G.C., M.C., C.B., L.P., M.T., I.C., S.P., D.S. and M.A. reviewed and edited the written work.

Funding
Open access funding provided by Umea University. This work has been partially funded by project PE0000013-FAIR of the Ministry of University and Research.

Data availability
Data will be provided upon request to the corresponding author.

Declarations

Ethics approval and consent to participate
Informed consent to participate was obtained from all of the participants in the study and all data were managed in accordance with the GDPR regulation. The study was approved by the Fondazione IRCCS Ca' Granda Ospedale Maggiore Policlinico ethical committee, to which our center refers (protocol ID: 2417, approved on September 29, 2021).

Consent for publication
Not applicable.

Competing interests
The authors declare no competing interests.

Received: 14 June 2024 Accepted: 25 October 2024
Published online: 27 November 2024

References

1. WHO. WHO Coronavirus (COVID-19) Dashboard. 2020. https://covid19.who.int/. Accessed 3 May 2023.
2. Fiscon G, Salvadore F, Guarrasi V, Garbuglia AR, Paci P. Assessing the impact of data-driven limitations on tracing and forecasting the outbreak dynamics of COVID-19. Comput Biol Med. 2021;135:104657.
3. Del Rio C, Collins LF, Malani P. Long-term health consequences of COVID-19. JAMA. 2020;324(17):1723–4.
4. Soriano JB, Murthy S, Marshall JC, Relan P, Diaz JV. A clinical case definition of post-COVID-19 condition by a Delphi consensus. Lancet Infect Dis. 2022;22(4):e102–7.
5. Carfì A, Bernabei R, Landi F. for the Gemelli Against COVID-19 Post-Acute Care Study Group. Persistent Symptoms in Patients After Acute COVID-19. JAMA. 2020;324(6):603-5.
6. Bailey J, Lavelle B, Miller J, Jimenez M, Lim PH, Orban ZS, et al. Multidisciplinary Center Care for Long COVID Syndrome–a Retrospective Cohort Study. Am J Med. 2023. https://doi.org/10.1016/j.amjmed.2023.05.002.

7. Duong-Quy S, Vo-Pham-Minh T, Tran-Xuan Q, Huynh-Anh T, Vo-Van T, Vu-Tran-Thien Q, et al. Post-COVID-19 Pulmonary Fibrosis: Facts—Challenges and Futures: A Narrative Review. Pulm Ther. 2023;9:1–13.
8. Moreno-Pérez O, Merino E, Leon-Ramirez JM, Andres M, Ramos JM, Arenas-Jiménez J, et al. Post-acute COVID-19 syndrome. Incidence and risk factors: A Mediterranean cohort study. J Infect. 2021;82(3):378–83.
9. Post COVID-19 condition (Long COVID). https://www.who.int/europe/news-room/fact-sheets/item/post-covid-19-condition. Accessed 7 Dec 2022.
10. Cau R, Faa G, Nardi V, Balestrieri A, Puig J, Suri JS, et al. Long-COVID diagnosis: from diagnostic to advanced AI-driven models. Eur J Radiol. 2022;148:110164.
11. Morante-García W, Zapata-Boluda RM, García-González J, Campuzano-Cuadrado P, Calvillo C, Alarcón-Rodríguez R. Influence of social determinants of health on COVID-19 infection in socially vulnerable groups. Int J Environ Res Public Health. 2022;19(3):1294.
12. Coronavirus Disease 2019 (COVID-19): A Systematic Review of Imaging Findings in 919 Patients. https://www.ajronline.org/doi/10.2214/AJR.20.23034. Accessed 14 Mar 2020.
13. Rofena A, Guarrasi V, Sarli M, Piccolo CL, Sammarra M, Zobel BB, et al. A deep learning approach for virtual contrast enhancement in contrast enhanced spectral mammography. Comput Med Imaging Graph. 2024;116:102398.
14. Mano LY, Torres AM, Morales AG, Cruz CCP, Cardoso FH, Alves SH, et al. Machine Learning Applied to COVID-19: A Review of the Initial Pandemic Period. Int J Comput Intell Syst. 2023;16(1):73–24. https://doi.org/10.1007/s44196-023-00236-3.
15. Dogan O, Tiwari S, Jabbar MA, Guggari S. A systematic review on AI/ML approaches against COVID-19 outbreak. Complex Intell Syst. 2021;7(5):2655–78. https://doi.org/10.1007/s40747-021-00424-8.
16. Guarrasi V, Tronchin L, Albano D, Faiella E, Fazzini D, Santucci D, et al. Multimodal explainability via latent shift applied to COVID-19 stratification. Pattern Recognit. 2024;156:110825.
17. Antony B, Blau H, Casiraghi E, Loomba JJ, Callahan TJ, Laraway BJ, et al. Predictive models of long COVID. EBioMedicine. 2023;96:51–64.
18. Tang CY, Gao C, Prasai K, Li T, Dash S, McElroy JA, et al. Prediction models for COVID-19 disease outcomes. Emerg Microbes Infect. 2024;(just-accepted):2361791.
19. Islam MN, Islam MS, Shourav NH, Rahman I, Faisal FA, Islam MM, et al. Exploring post-COVID-19 health effects and features with advanced machine learning techniques. Sci Rep. 2024;14(1):9884.
20. Pfaff ER, Girvin AT, Bennett TD, Bhatia A, Brooks IM, Deer RR, et al. Identifying who has long COVID in the USA: a machine learning approach using N3C data. Lancet Digit Health. 2022;4(7):e532–41.
21. Kessler R, Philipp J, Wilfer J, Kostev K. Predictive Attributes for Developing Long COVID–A Study Using Machine Learning and Real-World Data from Primary Care Physicians in Germany. J Clin Med. 2023;12(10):3511.
22. Sudre CH, Murray B, Varsavsky T, Graham MS, Penfold RS, Bowyer RC, et al. Attributes and predictors of long COVID. Nat Med. 2021;27(4):626–31.
23. Etienne M, Ettalhaoui N, Everding AG, Evers M, Fabre I, Fabre M, et al. ISARIC-COVID-19 dataset: a prospective, standardized, global dataset of patients hospitalized with COVID-19. Sci Data. 2022;9(1):454.
24. Alarcón-Rodríguez J, Fernández-Velilla M, Ureña-Vacas A, Martín-Pinacho J, Rigual-Bobillo J, Jaureguízar-Oriol A, et al. Radiological management and follow-up of post-COVID-19 patients. Radiol (Engl Ed). 2021;63(3):258–69.
25. Yang W, Yan F. Patients with RT-PCR-confirmed COVID-19 and normal chest CT. Radiology. 2020;295(2):E3–E3.
26. EpiCentro - Epidemiology for public health. https://www.epicentro.iss.it. Accessed 4 Jan 2024.
27. Solomon JJ, Heyman B, Ko JP, Condos R, Lynch DA. CT of post-acute lung complications of COVID-19. Radiology. 2021;301(2):E383–95.
28. Singh KN, Mantri JK. Clinical decision support system based on RST with machine learning for medical data classification. Multimed Tools Appl. 2024;83(13):39707–30. https://doi.org/10.1007/s11042-023-16802-y.
29. Xing W, Bei Y. Medical Health Big Data Classification Based on KNN Classification Algorithm. IEEE Access. 2019;8:28808–19. https://doi.org/10.1109/ACCESS.2019.2955754.
30. Huang S, Cai N, Pacheco PP, Narrandes S, Wang Y, Xu W. Applications of Support Vector Machine (SVM) Learning in Cancer Genomics. Cancer Genomics Proteomics. 2018;15(1):41–51. https://doi.org/10.21873/cgp.20063.
31. Zhang C, Ma Y. Ensemble machine learning: methods and applications. Springer; 2012.
32. Guarrasi V, Soda P. Multi-objective optimization determines when, which and how to fuse deep networks: An application to predict COVID-19 outcomes. Comput Biol Med. 2023;154:106625.
33. Guarrasi V, D'Amico NC, Sicilia R, Cordelli E, Soda PA, multi-expert system to detect covid-19 cases in x-ray images. In: 2021 IEEE 34th International Symposium on Computer-Based Medical Systems (CBMS). IEEE; 2021. pp. 395–400.
34. Guarrasi V, Soda P. Optimized fusion of CNNs to diagnose pulmonary diseases on chest X-Rays. In: International Conference on Image Analysis and Processing. Springer; 2022. pp. 197–209.
35. Cruz RM, Sabourin R, Cavalcanti GD. Dynamic classifier selection: Recent advances and perspectives. Inf Fusion. 2018;41:195–216.
36. Guarrasi V, D'Amico NC, Sicilia R, Cordelli E, Soda P. Pareto optimization of deep networks for COVID-19 diagnosis from chest X-rays. Pattern Recogn. 2022;121:108242.
37. Soui M, Mansouri N, Alhamad R, Kessentini M, Ghedira K. NSGA-II as feature selection technique and AdaBoost classifier for COVID-19 prediction using patient's symptoms. Nonlinear Dyn. 2021;106(2):1453–75. https://doi.org/10.1007/s11071-021-06504-1.
38. Ramón A, Torres AM, Milara J, Cascón J, Blasco P, Mateo J. eXtreme Gradient Boosting-based method to classify patients with COVID-19. J Invest Med. 2022;70(7):1472–80. https://doi.org/10.1136/jim-2021-002278.
39. Guyon I, Weston J, Barnhill S, Vapnik V. Gene selection for cancer classification using support vector machines. Mach Learn. 2002;46:389–422.
40. Breiman L. Random Forests. Mach Learn. 2001;45(1):5–32. https://doi.org/10.1023/A:1010933404324.
41. Duin RP. The combining classifier: to train or not to train? In: 2002 International Conference on Pattern Recognition. vol. 2. IEEE; 2002. pp. 765–770.
42. Cordella LP, Foggia P, Sansone C, Tortorella F, Vento M. Reliability parameters to improve combination strategies in multi-expert systems. Pattern Anal Applic. 1999;2:205–14.
43. Iannello G, Percannella G, Sansone C, Soda P. On the use of classification reliability for improving performance of the one-per-class decomposition method. Data Knowl Eng. 2009;68(12):1398–410.
44. Stahlschmidt SR, Ulfenborg B, Synnergren J. Multimodal deep learning for biomedical data fusion: a review. Brief Bioinform. 2022;23(2):bbab569.
45. Ramachandram D, Taylor GW. Deep multimodal learning: A survey on recent advances and trends. IEEE Signal Proc Mag. 2017;34(6):96–108.
46. Caruso CM, Guarrasi V, Cordelli E, Sicilia R, Gentile S, Messina L, et al. A multimodal ensemble driven by multiobjective optimisation to predict overall survival in non-small-cell lung cancer. J Imaging. 2022;8(11):298.
47. Borisov V, Leemann T, Seßler K, Haug J, Pawelczyk M, Kasneci G. Deep neural networks and tabular data: a survey. IEEE Trans Neural Netw Learn Syst. 2022;35(6):7499–519.
48. Ruffini F, Tronchin L, Wu Z, Chen W, Soda P, Shen L, et al. Multi-Dataset Multi-Task Learning for COVID-19 Prognosis. 2024. arXiv preprint arXiv:2405.13771.
49. Yong SJ, Liu S. Proposed subtypes of post-COVID-19 syndrome (or long-COVID) and their respective potential therapies. Rev Med Virol. 2022;32(4):e2315.

Publisher's Note

Springer Nature remains neutral with regard to jurisdictional claims in published maps and institutional affiliations.
